# Molded Round Window Niche Implant as a Dexamethasone Delivery System in a Cochlear Implant-Trauma Animal Model

**DOI:** 10.3390/pharmaceutics16091236

**Published:** 2024-09-23

**Authors:** Chunjiang Wei, Ziwen Gao, Robert Mau, Thomas Eickner, Gabor Jüttner, Nicklas Fiedler, Hermann Seitz, Thomas Lenarz, Verena Scheper

**Affiliations:** 1 Department of Otolaryngology, Hannover Medical School, Carl-Neuberg-Str. 1, 30625 Hannover, Germany; Wei.Chunjiang@mh-hannover.de (C.W.); gao_ziwen@fudan.edu.cn (Z.G.);; 2 Cluster of Excellence “Hearing4all”, German Research Foundation (DFG; “Deutsche Forschungsgemeinschaft”), Hannover Medical School, Carl-Neuberg-Str. 1, 30625 Hannover, Germany; 3 Lower Saxony Center for Biomedical Engineering, Implant Research and Development (NIFE), Stadtfelddamm 34, 30625 Hannover, Germany; 4 ENT Institute and Department of Otorhinolaryngology, Eye & ENT Hospital, Fudan University, Shanghai 200031, China; 5 Microfluidics, Faculty of Mechanical Engineering and Marine Technology, University of Rostock, Justus-von-Liebig Weg 6, 18059 Rostock, Germany; 6 Institute for Biomedical Engineering, University Medical Center Rostock, University of Rostock, Friedrich-Barnewitz Straße 4, 18119 Rostock, Germany; 7 Kunststoff-Zentrum in Leipzig gGmbH, Erich-Zeigner-Allee 44, 04229 Leipzig, Germany; 8 Department Life, Light & Matter, Interdisciplinary Faculty, University of Rostock, Albert-Einstein-Str. 25, 18059 Rostock, Germany

**Keywords:** local inner ear drug therapy, round window niche implant, cochlear implant surgery, dexamethasone, guinea pig, local pharmacotherapy

## Abstract

Background: Preserving residual hearing after cochlear implant (CI) surgery remains a crucial challenge. The application of dexamethasone (DEX) has been proven to positively affect residual hearing. To deliver DEX in a localized and controlled way, a round window niche implant (RNI), allowing drug diffusion via the round window membrane into the cochlea, may be used. To prove this concept, an RNI for guinea pigs as a CI-trauma model was manufactured by molding and tested for its drug release in vitro and biological effects in vivo. Methods: The RNIs were molded using silicone containing 10% DEX. Release was analyzed over time using high-performance liquid chromatography (HPLC). Fourteen adult guinea pigs were randomly assigned to two groups (CI or CI + RNI group). All animals received a unilateral CI electrode insertion trauma followed by CI insertion. The CI + RNI group was additionally implanted with an RNI containing 10% DEX. Animals were followed up for 4 weeks. Acoustically evoked auditory brainstem response and impedance measurement, micro-computed tomography (µCT) imaging, and histology were performed for evaluation. Results: DEX was released for more than 250 days in vitro, with an initial burst followed by a slower release over time. Comparing the hearing threshold shift (from day 0 to day 28) of the CI and CI + RNI groups, significant differences were observed at 32 and 40 kHz. The impedance shift at basal contacts was lower in the CI + RNI group than in the CI group. Moreover, the fibrosis in the lower basal turn was reduced in the CI + RNI group in contrast to the CI group. Conclusions: The RNI containing 10% DEX has anti-inflammatory potential concerning fibrosis inhibition and has beneficial effects on hearing preservation at high frequencies.

## 1. Introduction

The preservation of residual hearing stands as a pivotal factor influencing the efficacy of cochlear implant (CI) surgeries. The main factors contributing to residual hearing include age, electrode array characteristics, surgical techniques, inflammation, and fibrosis [1,2,3,4,5,6,7]. After cochlear implantation, the surgical trauma often results in an acute inflammatory response. Subsequently, a chronic inflammatory reaction may occur due to the foreign body response, leading to fibrotic reaction and new bone formation [8,9,10]. This process contributes to increased impedance and diminished efficiency of electrical stimulation [11,12,13]. Moreover, research indicates that the severity of fibrotic reactions and new bone formation is notably pronounced around the cochleostomy, which is expected to change the movement of the apical basilar membrane, thereby affecting the residual low-frequency hearing [7]. Therefore, reducing the inflammatory response to CI surgery is important for preserving residual hearing.

Many studies have demonstrated the beneficial effects of steroids such as dexamethasone (DEX), methylprednisolone, prednisolone, and triamcinolone in CI surgery [14,15,16]. Among these, DEX, a glucocorticoid well known for its anti-inflammatory effects [17], is a commonly used compound in CI therapy and was used in this study as a model drug to enable the discussion of our results with the various published studies on DEX in relation to CI [18]. The released DEX in perilymph could inhibit the release and expression of inflammation-related factors [17], such as macrophages, neutrophils, TNF-α, IL-1, prostaglandins, and leukotrienes. Additionally, it modulates immune function by inhibiting the activity of T and B lymphocytes, thereby alleviating immune-mediated inflammatory responses and reducing the risk of foreign body reactions and fibrosis following cochlear implantation surgery. Furthermore, DEX could also help against stress-induced programmed hair cell death within the cochlea [19,20], ultimately helping to preserve residual hearing.

The administration of DEX before, during, and following cochlear implantation still lacks standardization through intravenous injection or oral administration [21], which is easy to apply and less invasive. Nevertheless, the limited permeability of drugs across the blood–labyrinth barrier (BLB) necessitates high doses of DEX, which may increase the risks of adverse effects, like the inhibition of the hypothalamic–pituitary axis [22,23]. Given the prolonged inflammatory responses post-cochlear implant surgery, a sustained application of DEX appears necessary [14]. To overcome the issues of systemic delivery, local drug delivery has been widely investigated. One study demonstrated that local DEX delivery can reduce intracochlear inflammation and better preserve residual hearing compared with the systemic administration of the drug [24].

**Figure 1:**
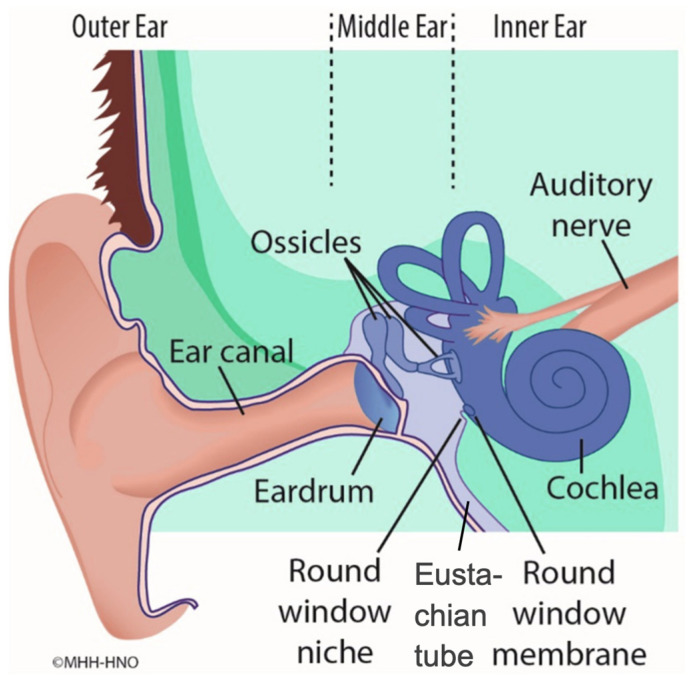
An anatomy diagram of the outer ear, the middle ear and the inner ear. The middle ear contains the ossicles and is filled with air. It is connected to the fluid-filled cochlea via the round window niche and the round window membrane.

Local inner ear drug delivery primarily contains intratympanic, intralabyrinthine, and intracochlear delivery. Intratympanic delivery, in particular, offers a less invasive route, whereby drugs diffuse from the air-filled and mucosa-lined middle ear across the round window membrane (RWM) (Figure 1) into the fluid-filled scala tympani of the cochlea. The RWM is an epithelial barrier that consists of three layers. The first and outer layer, facing the middle ear, consists of epithelial cuboidal cells that form tight junctions. The tight junctions prevent the passage of most molecules via passive diffusion. The second layer is made of fibroblasts, collagen, and elastic fibers and contains blood and lymph vessels, as well as nerve endings. The third layer consists of squamous flat inner epithelial cells facing the scala tympani [25]. The scala tympani is a relatively enclosed chamber filled with perilymph, facilitating the delivery of drugs to target cells within the inner ear, including hair cells, supporting cells, and spiral ganglion neurons. The perilymph is an extracellular fluid with a composition similar to cerebrospinal fluid (CSF) and characterized by a high sodium concentration (~140 mM) and low levels of potassium (~5 mM) and calcium (~1.2 mM) [26]. 

The drug delivery systems used to apply the drugs through intracochlear or intratympanic administration include hydrogels, nanoparticles, controlled-release formulations, and, in the past, pump-based delivery. Hydrogels, such as those based on poloxamer [27] or hyaluronic acid [28], provide a sustained release of drugs at the application site, enhancing therapeutic efficacy. Nanoparticles, often composed of biodegradable polymers like PLGA, offer the advantage of targeted delivery and prolonged drug release [29,30]. Controlled-release systems, including drug-eluting cochlear implants, are being explored to provide continuous drug delivery over extended periods, which is particularly beneficial for chronic conditions like Meniere’s disease or sensorineural hearing loss [31,32,33]. These systems aim to overcome the limitations of systemic drug delivery by ensuring higher local drug concentrations while minimizing systemic side effects. Intracochlear delivery involves the direct administration of therapeutic agents into the cochlea, often through cochleostomy or round window membrane injection. This method allows for precise drug distribution but is invasive and generally reserved for severe cases. Intratympanic application is less invasive and more commonly used in clinical practice. However, intratympanic drug delivery continues to face various challenges, like obstruction of the RWM in up to 60% of patients [34,35,36], the restrictions of the RWM on molecular penetration, potential drug loss through the Eustachian tube [37,38], and uncontrolled drug load of matrices and unknown pharmacokinetics of most drugs. 

To overcome most of the previously mentioned points, a drug-eluting implant based on the round window niche (RWN) anatomy was designed: the round window niche implant (RNI) [39]. This implant can be well positioned within the RWN to facilitate drug diffusion through the RWM into the cochlea. For CI patients, the sustained local delivery of DEX to the inner ear could effectively mitigate acute inflammation resulting from surgery, as well as trauma related to electrode insertion and chronic inflammation induced by the prolonged presence of the electrode, which can lead to residual hearing loss. This localized approach also avoids the adverse effects commonly associated with extended high-dose systemic DEX administration [32]. To account for the patient-specific anatomy of the niche [40], RNIs should be built, enabling a free-form concept, for example, through additive manufacturing. Such a 3D-printed RNI containing 1% DEX was investigated in a CI-trauma animal model [39]. The findings revealed that employing RNI with 1% DEX led to a significant decrease in fibrotic development. However, its efficacy in preserving residual hearing remained uncertain. A possible explanation could be an inadequate, i.e., too low, DEX load of the RNI. Given the absence of valid data or even standardized guidelines recommending the optimal DEX concentrations to preserve residual hearing in CI surgery, further study is needed to identify effective but safe dosages. Previous investigations from our group involved the evaluation of RNIs with varying DEX concentrations (1%, 5%, 10%, and 20%) in vitro, demonstrating their biocompatibility and bio-efficacy [39]. However, it was noted that the RNI containing 20% DEX exhibited a decrease in material hardness, which may affect its long-term stability. Therefore, in this study, we selected the 10% DEX-loaded RNI as the candidate to investigate the efficacy of a higher DEX load in the RNI. 

## 2. Materials and Methods

### 2.1. RNI Preparation 

**Figure 2:**
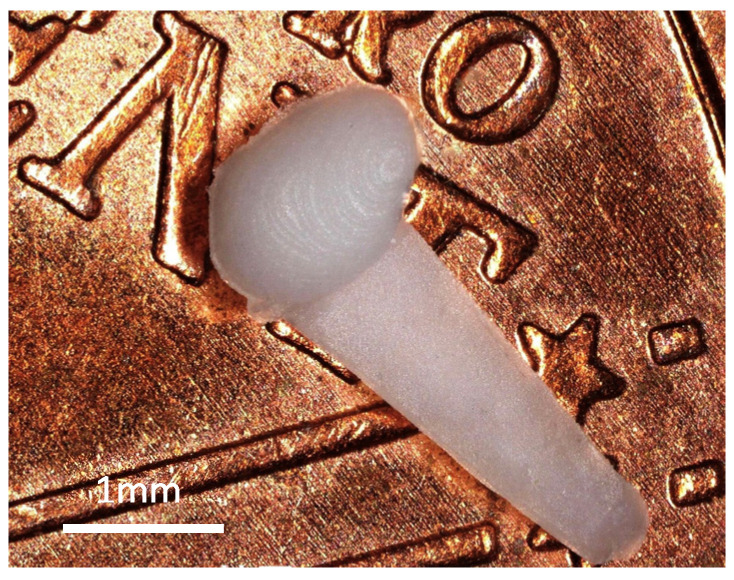
A molded RNI containing 10% DEX consists of the RWN part and a handle.

RNIs containing 10% DEX were designed and manufactured as previously described [41]. In short, the dimensions of the round window niche part of the implant were ~1.30 mm × 0.95 mm × 0.60 mm and were equipped with a handle (~3.00 mm × 1.00 mm × 0.30 mm; Figure 2). Medical-grade silicone elastomer MED-4244 (NuSil Technology LLC, Radnor, PA, USA) containing 10 wt% DEX (base, powder, CAS-Nr. 50-02-2, Sanofi SA, Paris, France) was used. MED-4244 is a low-consistency, pourable, and translucent silicone elastomer composed of a silicone base (part A) and a curing agent (part B), which are mixed in a 10:1 weight ratio. The elastomer cures with heat via addition-cure chemistry. The silicone-DEX mixture was provided by MedEl Corp., Austria. It is already used in pre-clinical studies investigating DEX eluting cochlear implants [42]. The silicone-DEX mixture was injected into a mold using the micro injection molding machine formicaPlast (Klöckner DESMA Elastomertechnik GmbH, Achim, Germany). The mold was 3D-printed via the high-resolution printer Asiga Pro 4K45 (Asiga, Alexandria, Australia) using resin Asiga PlasGRAY V2 (Asiga, Alexandria, Australia). 

### 2.2. In Vitro Drug Release Test

The masses of three RNI were determined on a Kern 770 micro-balance (KERN & Sohn, Balingen, Germany). Subsequently, the RNIs were placed in 4 mL glass vials and stored at 37 °C in 2 mL isotonic saline (0.9% NaCl, B.Braun, Melsungen, Germany) on a lab shaker (Heidolph, Schwabach, Germany) at 100 rpm. For sampling, the medium was exchanged completely after defined time periods (h): 0.25; 0.75; 1.5; 3; 6; 13; 29; 101; 197; 317; and then every 7 days for an additional 33 weeks. The sampled medium was diluted 1:1 with Methanol (Carl Roth, Karlsruhe, Germany)/dest. Water (Ultrapure water system (Sartorius, Göttingen, Germany)) prior to the high-performance liquid chromatography (HPLC) measurements. Quantification of DEX was performed on an HPLC system (Knauer Wissenschaftlicher Gerätebau, Dr. Ing. Herbert Knauer GmbH, Berlin, Germany) equipped with a Chromolith FastGrad RP-18e 50-2 column (Merck KGaA, Darmstadt, Germany). Methanol/Water 1:1 was used as a mobile phase in an isocratic HPLC method at a flow rate of 0.8 mL/min. Detection occurred with a UV-Detector at the wavelength λ = 254 nm [43]. For calibration, DEX standards with concentrations of 0.1, 0.5, 1.0, 2.0, 5.0, 10, and 50 µg/mL were used. 

### 2.3. Animals

Fourteen adult Dunkin-Hartley Guinea Pigs (Charles River Laboratories, France), weighing between 300 and 350 g, were utilized in this study. All experiments strictly adhered to the guidelines outlined in the German “Law on Protecting Animals” and the European Communities Council Directive 2010/63/EU, which govern the ethical use of animals for research purposes. This study was conducted with explicit approval from the local authorities (Lower Saxony State Office for Consumer Protection and Food Safety (LAVES), Oldenburg, Germany, registration number 20/3592).

Prior to the experiments, all animals underwent a two-week acclimatization period within the animal facility. They were housed under meticulously controlled conditions, including regulated temperature and humidity levels. A consistent 24 h light-dark cycle (14 h light, 10 h dark) was maintained to establish standardized living conditions. Throughout the study duration, the animals were provided ad libitum access to both food and water to ensure their well-being.

All guinea pigs were randomly allocated into two groups: the CI group (n = 7) and the CI + RNI group (n = 7). All guinea pigs were unilaterally (right ear) implanted via cochleostomy with a CI. The CI group received only a CI, while guinea pigs in the CI + RNI group received the CI and an RNI containing 10% DEX.

### 2.4. Experimental Timeline

**Figure 3:**
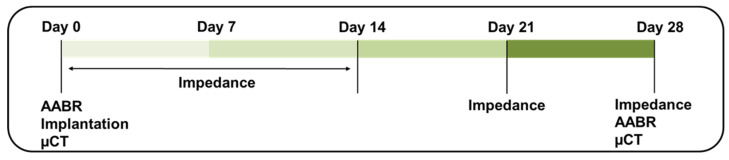
The timeline of measurements and interventions.

On day 0, the frequency-specific hearing thresholds of guinea pigs were initially assessed. Only guinea pigs exhibiting normal hearing thresholds were considered eligible for inclusion in the experiments. Subsequent to surgical interventions, a micro-computed tomography (µCT) scan was employed to verify the positions of the CI or the success of RNI insertion. Impedance measurements were conducted on day 0 post-operation and on days 1 through 14, as well as on day 21 and day 28, without anesthesia. Additionally, on day 28, the frequency-specific hearing thresholds were measured, and µCT scans were performed to assess the position of the CI and RNI. Subsequently, the guinea pigs were euthanized, and the cochleae were harvested for histological examination (Figure 3).

### 2.5. Surgery

Following anesthesia with intramuscular medetomidinhydrochloride (0.2 mg/kg), midazolam (1 mg/kg) and fentanyl (0.025 mg/kg) [44], animals were placed on a heating mat at 38 °C. The skin area to be incised received local anesthesia with prilocaine (Xylonest 1%, Aspen Germany GmbH, 0.5 mL, subcutaneous injection) [44]. The bulla was opened via a retroauricular approach, and the defect was enlarged to achieve optimal visualization of the RWN. 

**Figure 4:**
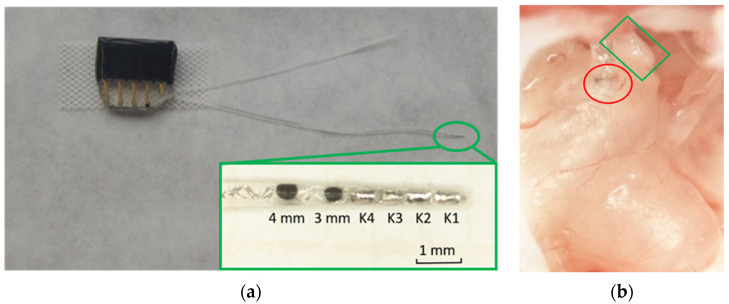
(**a**) Cochlear implant for guinea pig. The green box shows an enlarged view of the electrode array tip, with the positions of two black markers and the four platinum contacts marked, respectively. (**b**) Image of a cochlea with a focus on the basal region 28 days after implantation. The inserted RNI (green rectangle) and CI (red circle) are visible. The RNI was placed in the RWN while the CI was inserted in a cochleostomy 2 mm below the round window. The black marker illustrating a 4 mm distance from the electrode tip is visible. The electrode is cut for better handling and reduction of the danger of explantation during the process of tissue harvesting.

The cochlear implant (GP-Electrode, MED-EL GmbH, Innsbruck, Austria) utilized in guinea pigs comprised a connector, an electrode array, and a reference electrode. The tip of the electrode array featured four platinum contacts (K1, K2, K3, and K4 from apical to basal), with two black dots marking the distances at 3 and 4 mm from the tip. The diameter of the electrode array tip was approximately 300 µm (Figure 4a). The connector of the CI was securely affixed to the skull using two screws, UV-cement (Tetric EvoFlow^®^, Ivoclar Vivadent, Ellwangen, Germany), and Paladur (Paladur^®^, Kulzer GmbH, Hanau, Germany) [45]. The electrodes were guided through the skin and muscles to the vicinity of the bulla. A 0.7 mm hole was slowly drilled (AccuPen 6V+; RISystem AG, Landquart, CH) in the cochlea, positioned 2 mm below the round window (Figure 4b). To induce fibrosis, the electrode array was inserted thrice up to the second black point of the electrode (4 mm) [46]. Only animals of the CI + RNI group subsequently received an RNI. The RNI was positioned on the RWM, followed by careful adjustment using a probe to ensure correct placement of the RNI main body in the RWN (Figure 4b). Subsequently, UV-cement was used to fix the handle of the bony cochlear wall and close the bullostomy.

The reference electrode was positioned above the bulla defect within the musculature. The retroauricular incision was closed in two layers. Anesthesia antagonization was performed using medication that had been previously published [44]. Subsequently, the guinea pig was placed under red light until fully awakened and capable of maintaining its body temperature.

### 2.6. Acoustically Evoked Auditory Brainstem Response (AABR) Measurement

The AABR measurements were performed under anesthesia in an acoustically shielded chamber, progressing from the right ear to the left ear. The tests were facilitated using a Tucker-Davis Technology (TDT) System (Alachua, FL, USA) in conjunction with BioSigRP software (version 102). Sound was presented via a calibrated speaker (DT48, BeyerDynamic, Heilbronn, Germany) connected to the ear through a plastic cone. Acoustic tone stimuli (duration: 6 ms with 2 ms rising/falling ramps) with frequencies of 1, 2, 4, 8, 16, 32, and 40 kHz were generated and presented at sound pressure levels (SPLs) from 0 dB in 5 dB steps up to the SPL where a clear AABR wave was visible. The AABR signals were recorded using subdermal needle electrodes (CareFusion Nicolet, Middleton, WI, USA), including a positive electrode, reference electrodes, and a ground electrode. The signals were amplified, band-pass filtered, and recorded at a sampling rate of 100 kHz. Hearing thresholds were defined as the lowest stimulus required to evoke a visually replicable AABR waveform. Only animals with initial normal hearing (thresholds of ≤50 dB SPL) were included in this study.

### 2.7. Impedance Measurement

The system used for impedance measurement contained a MAX-Box and MAESTRO software version 8.0 (both MED-EL GmbH, Innsbruck, Austria). After fixation of the guinea pig with a soft towel, the CI was connected to the system by inserting the cable into the CI connector on the guinea pig’s head. Following this, the Impedance Field Telemetry (IFT) task was activated in monopolar mode. More details had been reported in a previous publication [45]. 

### 2.8. µCT Scan

Directly after implantation and on day 28, µCT scans were performed on anesthetized guinea pigs using an XtremeCTII system (ScancoMedical AG, Brüttisellen, Switzerland). The scan parameters were set to 1470 µA, 100 W, with an integration time of 90 ms and a resolution of 17 µm. The acquired data were converted to DICOM (digital imaging and communications in medicine) and reconstructed using COMET software [47]. The RNI was identified based on the difference in grayscale values.

### 2.9. Histology

The preparation of the temporal bone proceeded in steps following established protocols detailed in a previous publication [39], including fixation, decalcification, and clearing. In short, fibrosis detection was performed using confocal laser scanning microscopy (CLSM). For this, the tissue has to be fixed (paraformaldehyde) and decalcified (EDTA, Sigma-Aldrich Chemie GmbH, Schnelldorf, Germany) for up to four weeks. The cochleae were dehydrated, and the refractive index adjustment was using methyl salicylate benzyl benzoate (MSBB) in ethanol. For CLSM, the cochlea was placed in pure MSBB. For imaging, a Leica SP8 laser scanning confocal microscope (Leica Microsystems GmbH, Wetzlar, Germany) equipped with a 10× magnification objective lens (HC PL Fluotar 10×/0.30 Dry, Fa. Leica) was used. During scanning, a laser with a wavelength of 492 nm was employed, and the Transmitted Light Detector channel was opened to facilitate visualization of the electrodes. The slices were generated with 20 µm intervals (z-stack on) at a scanning speed of 400 Hz, 5× line averaging and 3× frame averaging. Subsequently, the slices were 3D reconstructed using Leica Confocal software (LAS X Science Microscope Software; version LAS X 3.5.7.23225). The reconstruction allowed for the observation of fibrotic growth in the scala tympani, with scoring based on a ranking score (Table 1). For an accurate assessment, we subdivided the area where fibrotic growth can be observed into three regions, namely the lower basal turn (LB), middle basal turn (MB), and upper basal turn (UB).

### 2.10. Statistical Analysis

Statistical analyses were conducted using GraphPad Prism^®^ version 8.4.3 (GraphPad Prism Software Inc., La Jolla, CA, USA). Due to the electrode detachment or wrong insertion, one guinea pig from the CI group and two guinea pigs from the CI + RNI group were excluded from the analysis of hearing threshold and fibrotic growth, resulting in N = 6 and N = 5, respectively. The normal distribution of the data was assessed using the Shapiro–Wilk test. Hearing thresholds and fibrotic growth were analyzed utilizing unpaired *t*-tests or Mann–Whitney tests, selected based on the outcome of the normality test. *p*-values below 0.05 were considered to be statistically significant. 

For the impedance analysis, additional animals were excluded due to broken electrodes over time, leaving only three valid impedance measurements on day 28 in each group (CI and CI + RNI). Consequently, a comparative analysis between groups was not possible due to the small sample size, so only the mean and standard deviation (SD) were reported.

## 3. Results

### 3.1. Drug Release

**Figure 5:**
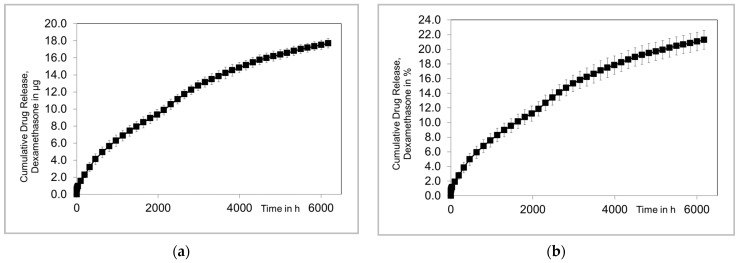

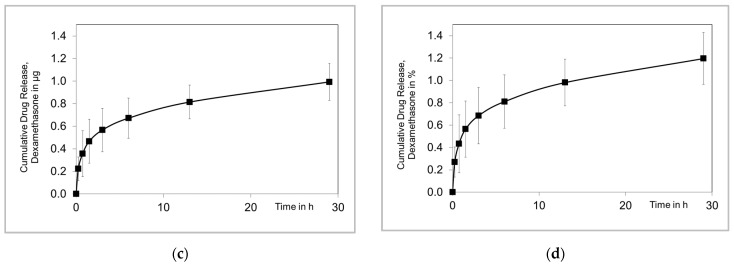
The diagrams show the cumulative drug release of DEX. (**a**) The absolute amount of released DEX within 35 weeks. (**b**) Relative DEX release in 35 weeks. (**c**) Enlarged representation of the absolute release over the first 29 h. (**d**) Enlarged representation of the relative release over the first 29 h. (**b**,**d**) were normalized to the calculated amount of DEX preload in the RNI based on the measured RNI mass.

The mean (±SD) of masses of the RNIs was 0.83 ± 0.02 mg. Based on the mean mass, the calculated mean DEX amount in the samples before incubation was 83.3 ± 2.5 µg (Table 2). The supernatant was sampled until 6000 h, i.e., 250 days, or 35 weeks, after the start of the incubation. The analysis still showed a measurable release at that time point. Therefore, the incubation was still running, and we report the data of the 250-day sampling period here (Figure 5). Data are reported as a cumulative release over time (Figure 5a) and released in relation to the amount of DEX at the time of manufacturing (Figure 5b). The release showed a two-phased progression. A burst release occurred within the first 29 h, during which a relatively large amount of DEX (ca. 1 µg in total) was released rather quickly (Figure 5c,d). This was followed by a phase in which the release tapers off. A release rate of about 0.6 µg/week was observed from the sixth week on; from week 19, the rate decreased to 0.2 µg/week. After 35 weeks, about 21% of the loaded DEX (total amount: 12.8 ± 0.6 µg) was released.

### 3.2. Hearing Threshold

**Figure 6:**
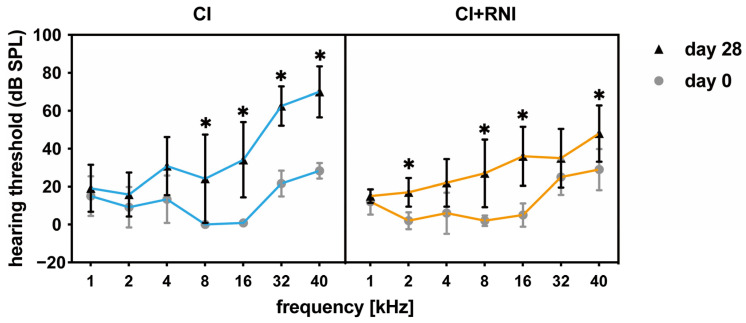
The frequency-specific hearing thresholds in the CI and CI + RNI groups on days 0 and 28 are depicted. A comparison between day 0 and day 28 reveals significant differences in the CI group at 8, 16, 32, and 40 kHz, while the CI + RNI group exhibits significant differences at 2, 8, 16, and 40 kHz. (* *p* < 0.05).

Compared with the baseline hearing threshold measurement on day 0, the hearing thresholds of the CI group were significantly increased at 8, 16, 32, and 40 kHz 28 days after CI surgery. In the CI + RNI group, significant differences compared with the baseline (day 0) are noted at frequencies of 2, 8, 16, and 40 kHz (*p* < 0.05), while no significant difference is observed at 32 kHz (Figure 6). Notably, the hearing threshold in the basal region of CI-only implanted animals was 62.5 ± 10.37 dB and 70 ± 13.42 dB at 32 and 40 kHz, whereas at the same frequencies in animals receiving an RNI in parallel to the CI, thresholds of 35 ± 15.41 dB and 48 ± 14.83 dB were measured at the same frequencies.

In addition, the not-implanted, contralateral ears of the CI group and CI + RNI group show no difference between day 0 and day 28 in frequency specific hearing thresholds (see Supplementary Material, Figure S1). 

**Figure 7:**
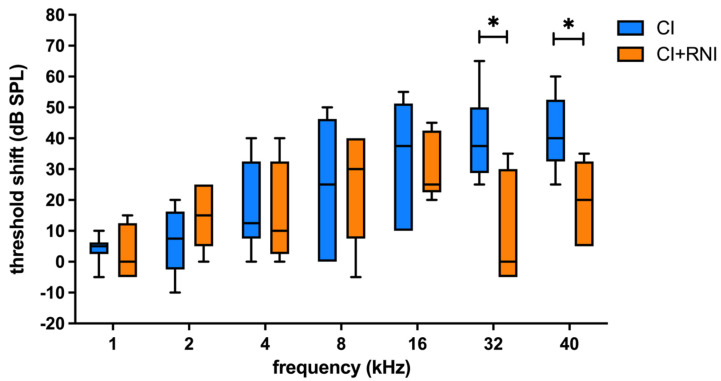
The frequency-specific threshold shifts (threshold change from day 0 to day 28) of the CI group and CI + RNI groups are plotted (median and min to max). Between the CI and CI + RNI groups, there are significant differences in threshold shifts at 32 and 40 kHz, with CI-only resulting in higher shifts. (* *p* < 0.05).

The hearing threshold shifts (difference from day 0 to day 28) of the cochlear implanted ears without an RNI decreased from the high to the low frequencies with 41.67 ± 12.52 dB at 40 kHz and 6.67 ± 10.8 dB at 2 kHz (Table 3). This trend was seen in CI + RNI-treated ears as well. Here, 19 ± 13.87 and 15 ± 10.61 dB were detected at 40 and 2 kHz, respectively. Comparing the hearing threshold shifts of the implanted side between the CI and CI + RNI group, significant differences were observed at 32 and 40 kHz, with the CI group suffering from increased thresholds compared with the CI + RNI group (Figure 7). 

### 3.3. Impedances

An overview of the impedance trends over time for each contact reveals a general pattern: initially, there was a slight decrease in impedance within the first three days, followed by a gradual increase, eventually stabilizing after 21 days. 

**Figure 8:**
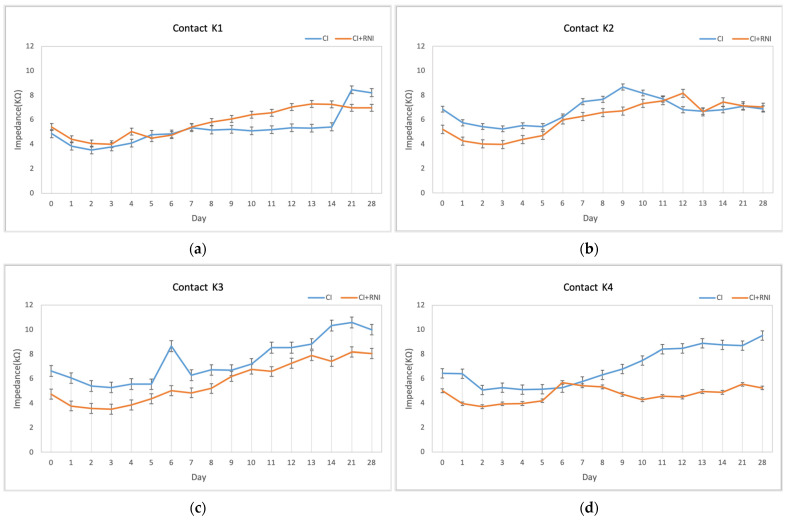
Clinical impedance measurements for both experimental groups—CI and CI + RNI—from day 0 to 14 and on days 21 and 28, separately illustrated from apical to basal for K1 (**a**), K2 (**b**), K3, (**c**) and K4 (**d**) are given. (**d**) is closest to the cochleostomy, (**a**) is the most apical contact.

At contact K1 (at the electrode tip), lower mean impedances were observed in the CI group than in the CI + RNI group at the end of the first two weeks (day 7: 5.3 kΩ versus 5.4 kΩ; day 14: 5.4 kΩ versus 7.2 kΩ). Notably, the CI + RNI group exhibited lower mean impedances than the CI group at the end of the last two weeks (day 21: 6.9 kΩ versus 8.4 kΩ; day 28: 6.9 kΩ versus 8.2 kΩ) (Figure 8a). The impedance shift from day 0 to day 28 increased more in the CI group than in the CI + RNI group (Table 4).

At contact K2, the impedance trends over time were comparable between the CI group and the CI + RNI group (Figure 8b), with the impedance shift increasing less in the CI group compared with the CI + RNI group (Table 4).

Lastly, at contacts K3 and K4 (more basal), the mean impedances in the CI + RNI group were lower compared with the CI group over the 28-day period (Figure 8c,d). At contact K3, the impedance shift in the CI group appeared similar to that of the CI + RNI group. However, at contact K4, the impedance shift was larger in the CI group (3 ± 2.5 kΩ) than in the CI + RNI group (0.3 ± 1.1 kΩ) (Table 4).

### 3.4. Histology 

**Figure 9:**
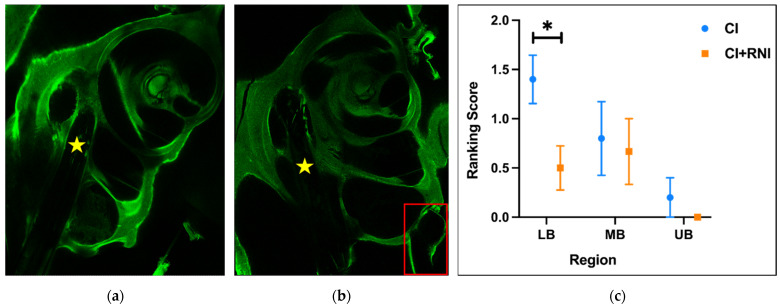
(**a**) Exemplary image of the lower basal turn of one cochlear implanted ear at 10× magnification (CI group; yellow star: electrode). (**b**) Image of the lower basal turn of a CI + RNI group animal at 10× magnification (yellow star: electrode; red rectangle: RNI). (**c**) Evaluation of fibrotic growth in different basal cochlea regions. In the most basal region, the lower basal turn (LB), the CI + RNI treatment resulted in significantly lower ranking scores compared with the CI group, while there were no differences between the two groups in the middle basal turn (MB) and upper basal turn (UB) (* *p* < 0.05).

Fibrotic tissue was observed in the basal turn of the cochlea (Figure 9a,b), in none of the cochleae extending toward more apical regions. In the CI group, from the lower basal turn (LB) to the middle basal turn (MB) to the upper basal turn (UB), the fibrosis ranking scores gradually decreased from 1.4 ± 0.35 (LB) to 0.8 ± 0.28 (MB) to 0.32 ± 0.14 (UB). A comparative analysis between the CI group and the CI + RNI group revealed less fibrous tissue growth in the LB in the CI + RNI group (0.5 ± 0.55) compared with the CI group (*p* < 0.05) and no fibrosis at all in the UB, as depicted in Figure 9c. In the MB and UB, there was no significant difference between the two groups (Figure 9c).

## 4. Discussion

The present study focused on investigating the safety and efficacy of a molded DEX containing silicone-based RNI in a cochlear implant animal model. In a previous approach, we already tested the biocompatibility, bio-efficacy, and drug release of the molded RNI, revealing that the molded RNI containing 10% DEX was biocompatible and bio-effective in vitro and exhibited sustained release of active DEX over a period exceeding 29 days [41]. The used silicone (Nusil MED-4244) is medical grade and can be considered for use in human implantation for a period of greater than 29 days [48]. The DEX used in our study (C22H29FO5, CAS No. 50-02-2; EC No: 200-003-9) has a water solubility of 89 ng/mL [49]. Saline has a higher solubility compared with water and PBS and also has a close similarity to perilymph in ionic concentration and tonicity. Since the RNI is connected to the RWM via body fluid eluted by the middle ear mucosa, the release is influenced by the composition of this fluid. Additionally, the perilymph affects the released DEX based on the respective solubility. Libau et al. report a DEX concentration in perilymph of 450 ng/mL [50], indicating that the solubility of DEX in perilymph is even higher.

The combination of both, DEX releasing silicone per se, was already investigated frequently by others, who reported a homogenous distribution of the DEX in the matrix, no swelling, a burst release, and a long-term drug elution [51,52]. The silicone-DEX composition used in this study differs from those studies but was already used in an in vitro test of cochlea implant functionalization using DEX-releasing silicone [42]. In that study, the authors studied the release rates in water, PBS, and saline and reported a burst release as seen for the molded RNI [41]. Additionally, the same silicone-DEX composition was used to prepare rods and to cochlear implant guinea pigs. Here, the release rate in perilymph was analyzed, and a burst release was observed as well [50]. 

To ascertain the complete profile of DEX release from the RNI, we extended the duration of the drug release test in the here presented study. The detected burst release is in line with our previous report [41] and other studies using different silicone elastomers as release matrices for DEX [46,51,53,54] and DEX sodium phosphate [55]. The release of DEX from the RNIs happens in a typical diffusion-controlled manner, showing a matrix system behavior [56,57]. The burst release occurs due to the surface near regions where the diffusion paths are short. Diffusion is a process that is dependent on the concentration gradient between the drug delivery system and the release medium, with a higher gradient leading to a faster diffusion. As the concentration gradient remains maximal at the beginning of the release, the total amount of released drug is higher within the first week. The second phase is characterized by a decreasing release rate as the drug concentration; thus, the concentration gradient in the implant also slowly decreases. The novelty of the reported data here is the finding that the molded RNI containing 10% DEX releases DEX for more than 6000 h, i.e., 250 days, or 35 weeks, at a relatively consistent amount, which is still detectable using HPLC. The detected amount of DEX within the burst release phase was ca. 1 µg in total. Within that phase (during the initial 29 h, e.g., seven time points), it showed the highest release during the first 15 min with 14.8 ng/mL/min, slowly decreasing to 0.19 ng/mL/min for the last 16 h. Hence, the average released amount per minute during the first 29 h was around 0.7 ng/mL/min. This initial release is higher than the effective dose range of 50 to 100 ng/mL suggested by a relevant study for the CI insertion trauma model [50]. During the remainder of the observation period, average DEX levels were about 300 ng/mL from the sixth week onward and 100 ng/mL from week 19 onward.

Despite various clinical trials investigating the effect of intratympanically applied steroids on hearing loss, there are no definitive data regarding the optimal DEX concentration for hearing protection following cochlear implantation. Hahn et al. reported that the DEX (DEX-dihydrogen phosphate disodium salt) delivery effectiveness was 1.5 ng/μL/min by continuously infusing high concentrations of DEX directly onto the RWM. However, the perilymph concentrations resulted in a very large difference (291 ± 369 ng/μL) [58]. Additionally, Du et al. demonstrated that 10% DEX nanoparticles with magnetic characteristics placed in the RWN were measured as delivering at 0.3 ng/μL/min [59], while the clinical data indicate the local level of DEX therapy was 5 μg/μL for one week [60,61], about 0.5 ng/μL/min. Moreover, measuring the actual release rate of a drug within the cochlea is challenging, as obtaining samples of inner ear fluid for drug concentration analysis may result in damage to the auditory structures, which is the reason why perilymph sampling until today is performed only in patients suffering from severe hearing loss. Consequently, the intracochlear drug release profile remains to be investigated in the future.

The residual hearing loss in CI recipients is influenced by several factors, including age, characteristics of the electrode array, and surgical techniques, as well as inflammation and fibrosis. When employing an electrode array for guinea pigs similar to those utilized in our study (hardness, diameter, and insertion depth), a notable threshold shift (25–30 dB) within the frequency range of 8 to 32 kHz has been reported [62]. Therefore, residual hearing loss observed in the CI group of our study (24, 33, and 40 dB at 8, 16, and 32 kHz, respectively; see Supplementary Data) is in line with the literature. Since no hearing loss was observed at the contralateral site, we can exclude that the hearing loss was due to age or exogenous noise exposure during housing. Together with the finding of fibrosis around all implanted CIs in the CI group, this indicates that the animal model of electrode insertion trauma applied in our study was successful. Taking into account the potential fluid exchange via the cochlea aqueduct from one ear to the other, it is possible that DEX from the implanted ear will migrate to the not-implanted ear. The AABR analysis of the hearing of not-implanted ears 28 days after RNI implantation showed no threshold deviation compared with baseline, indicating that the implantation of RNI containing 10% DEX has no effect on the hearing of the not-implanted contralateral ear.

By comparing the threshold shift within the measured frequencies of the CI and CI + RNI implanted ears, a significant difference was observed with reduced hearing loss, i.e., lower shifts, in the CI + RNI group at 40 and 32 kHz. This indicates the potential of RNI containing 10% DEX to rescue high-frequency hearing. 

In CI recipients, residual hearing typically persists at lower frequencies while the higher frequencies are already lost, which is an indication of cochlear implantation. Therefore, the evident protective effect on high-frequency hearing observed with an RNI containing 10% DEX is not relevant for preserving CI patient’s residual hearing yet. One reason for the missing effects in the lower frequencies may be the DEX diffusion along the cochlea turns. DEX penetrates into the cochlea via the RWM and spreads there through passive diffusion. A massive concentration gradient along the cochlea spiral has been shown for dexamethasone-phosphate [63]. Whether this also applies to the DEX used in our study and if such a concentration gradient may have had an impact on the not-detected hearing preservation at 16 kHz and lower frequencies can only be speculated. Since the problem of drug entry into the cochlea and distribution within the cochlea from the basal entry region toward the apical structures is a known fact [64], there are pre-clinical approaches to support the drug distribution along the spiral. Those approaches are, for example, focused on delivering the drug deep in the cochlea using micropumps [65] or CI-integrated catheters [66] or aiming to promote the distribution of basally applied drugs to the apical turns. Those approaches are, for example, magnetically driven nanoparticle-based drug guidance or steady streaming [67] and may be applicable to RNI-based drug delivery throughout the cochlea spiral in CI patients in the future. 

Next to directly to CI insertion-related hearing loss due to trauma or impact on normal inner ear mechanics, there are other acute and chronic processes after implantation leading to residual hearing loss. Electrode impedance reflects the resistance between the stimulating electrode and the return electrode, and alterations in the perilymph or extracellular fluid composition surrounding the electrodes, fibrous tissue, or new bone formation are expected to increase impedance [68,69,70,71]. A comparison of the average impedance shifts (from day 1 to day 28) between the experimental groups revealed that the shift observed in the basal region (K4) tended to be lower in the CI + RNI group. The reduction in impedance shift may be due to less fibrosis growth or fewer inflammatory factors adjacent to the contact K4 of the CI + RNI group. This finding is more descriptive since the limited sample size did not allow for statistical analysis.

While impedance measurements offer longitudinal but indirect insights into changes within the cochlea caused by a variety of factors, histological examination provides direct visualization of fibrous tissue at one specific time point. In our study, fibrosis was only detected in the basal turn, where the electrode was located. This is in line with previous reports where the tissue growth is limited to the electrode position [72]. In the cochlear implanted electrode insertion trauma control group (the CI group), the amount of fibrosis was the highest in the LB region and decreased toward the electrode tip. This pattern of fibrous growth predominantly surrounding the electrode, particularly at the cochleostomy side, had previously been reported [73]. Compared with the CI group, the fibrosis of CI + RNI-treated ears was significantly reduced in the lower basal region. No statistically relevant differences were observed in the more apical regions, but trends of reduced fibrosis in RNI-implanted ears were observed. This may be due to the reduced fibrosis per se (in controls), which leads to a reduction of the possible nominal difference between the two groups and subsequent limited ability to detect relevant treatment impacts. In other words, since the fibrosis is already relatively low in the middle and upper basal regions of the control group, the possible treatment effect of the RNI cannot be detected anymore. A second or parallel reason for the trend but not statistically relevant difference in fibrosis in the more apical regions may be the DEX diffusion along the cochlea turns. This has already been discussed in the previous paragraph.

The here presented data show a tremendously long—more than 35 weeks—DEX release out of the molded, silicone-made, initially 10% DEX containing RNI. In a CI-trauma model, the RNI implantation significantly reduced fibrosis and hearing loss in the high frequencies. Therefore, it seems as if the initial burst release of DEX is sufficient to treat acute inflammatory responses resulting from CI surgery. Future studies have to be conducted to investigate if a further increase in drug preload will result in even higher positive effects for CI recipients. 

Next to the acute effects, we propose that the RNI might be suitable for preventing long-term chronic inflammatory responses post-CI surgery as well. As already shown for other silicone elastomers and DEX formulations, the release duration may last for years [52]. This may be relevant for delayed pathological processes such as loss of residual hearing, which can occur more than 12 months after CI surgery. 

Since the biological effects in the here used animal model was restricted to the basal, high frequency region other inner ear pathologies where the high frequencies need to be treated, such as idiopathic sudden sensorineural hearing loss should be future targets for this local drug delivery system. 

The data are very promising, but for a translation of the approach of using a round window niche implant for local and sustained inner ear therapy to modulate the inner ear environment in CI patients, more pre-clinical work is needed. Future investigations should focus on optimizing the DEX load and concentration gradient for optimal drug application. Additionally, it is essential to determine whether variations in the effective contact surface area and implant shape contribute to differences in implant performance. The potential adverse effects of the RNI, such as impediments to round window membrane movement, which may affect the hearing threshold, should also be thoroughly investigated. The silicone used is non-degradable, potentially requiring subsequent surgery for removal. For patients, this may be a drawback, and therefore, future investigations may explore biodegradable materials to facilitate clinical acceptance of the RNI.

## 5. Conclusions

Until today, there has not been a safe and effective delivery system for the local application of drugs to the inner ear without opening the inner ear and thereby traumatizing its delicate structures. This study verified that an RNI containing 10% DEX can sustainably release DEX for more than 35 weeks in vitro. Additionally, this study is the first-ever reporting on the successful implantation and efficacy of a molded, drug-containing implant for the RNI and demonstrated its efficacy in mitigating fibrosis and preserving high-frequency hearing after CI surgery in an electrode insertion trauma animal model. Furthermore, the drug release for more than 35 weeks suggested that this implant holds significant potential for long-term therapeutic use. In future studies, other compounds addressing other cascades of inner ear pathologies may be encapsulated into the RNI, potentially enabling therapy for other inner ear diseases. Such an implant presents a promising option for patients in need of long-term, localized, and individualized pharmacotherapy of the inner ear. Future investigations are warranted to explore the broader effects of RNI on auditory function, including its impact on basilar membrane movement. Furthermore, the development of advanced methodologies to monitor in vivo drug release profiles over time will be essential to enhance the clinical applicability of this technology.

## Figures and Tables

**Table 1:** Ranking score for fibrotic growth in the cochlea.

Ranking Score	Fibrotic Growth
0	none
1	1 to 2 cell layers
2	thick cell layers
3	scala tympani filled up completely

**Table 2:** Masses and calculated drug load of the RNI samples prior to the drug release testing.

RNI	1	2	3
Mass (mg)	0.83	0.81	0.86
Calculated DEX amount (mg)	0.083	0.081	0.086

**Table 3:** Mean and standard deviation of frequency-specific hearing threshold shift (dB SPL).

	1 kHz	2 kHz	4 kHz	8 kHz	16 kHz	32 kHz	40 kHz
CI	4.17 ± 4.9	6.67 ± 10.8	17.5 ± 14.75	24.17 ± 23.33	33.33 ± 20.9	40 ± 14.14	41.67 ± 12.52
CI + RNI	3 ± 9.08	15 ± 10.61	16 ± 16.36	25 ± 18.71	31 ± 10.84	10 ± 18.71	19 ± 13.87

**Table 4:** Mean and standard deviation of impedance shifts (from day 0 to day 28) of contacts (kΩ).

	CI	CI + RNI
Contact K1	3.3 ± 4	1.6 ± 1.8
Contact K2	0 ± 0.2	1.8 ± 1.3
Contact K3	3.4 ± 5.8	3.3 ± 4.4
Contact K4	3 ± 2.5	0.3 ± 1.2

## Data Availability

The raw data supporting the conclusions of this article will be made available by the authors upon request.

## Supplementary Materials

The following supporting information can be downloaded at https://www.mdpi.com/article/10.3390/pharmaceutics16091236/s1, Figure S1: The frequency specific hearing thresholds (mean ± SD) on day 0 and day 28 of not-implanted ears in the CI and CI + RNI group.
