# Supplementary material for: Molded Round Window Niche Implant as a Dexamethasone Delivery System in a Cochlear Implant-Trauma Animal Model
Source: Pharmaceutics. 2024 Sep 23;16(9):1236. doi: 10.3390/pharmaceutics16091236 (PMC11434969; doi:10.3390/pharmaceutics16091236)
Supplement: Supplementary file 1 [file pharmaceutics-16-01236-s001.zip › Supplementary material-Figure S1.pdf]

Supplementary material

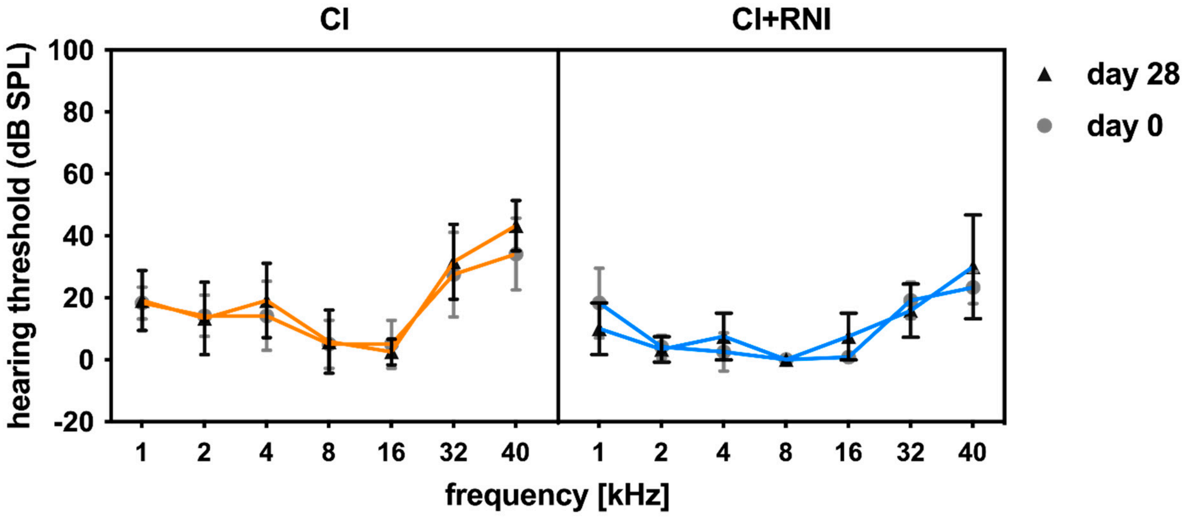

Figure S1. The frequency specific hearing thresholds (mean ± SD) on day 0 and day 28 of not-implanted ears in the CI and CI+RNI group are illustrated. There is no difference between day 0 and day 28 in both groups.
